# Prevalence of hypogammaglobulinemia after non-anti-CD20 therapies and impact of switching to rituximab/ocrelizumab in multiple sclerosis

**DOI:** 10.1016/j.neurot.2025.e00760

**Published:** 2025-10-04

**Authors:** Marine Perriguey, Camille Rigollet, Sean A. Freeman, Lisa Graille-Avy, Jean-Christophe Lafontaine, Bruno Lemarchant, Tifanie Alberto, Sarah Demortière, Clémence Boutiere, Audrey Rico, Frédéric Hilézian, Pierre Durozard, Jean Pelletier, Adil Maarouf, Hélène Zéphir, Bertrand Audoin

**Affiliations:** a Aix Marseille Univ, APHM, Hôpital de la Timone, Marseille, France; b Department of Neurology, CRC-SEP, CHU of Lille, Lille, France; c Univ. Lille, INSERM, CHU Lille, Laboratory of Neuroinflammation and Multiple Sclerosis (NEMESIS), U1172, Lille, France; d Aix Marseille Univ, CNRS, CRMBM, Marseille France; e Centre Hospitalier d’Ajaccio, France

**Keywords:** Multiple sclerosis, Ocrelizumab, Rituximab, Hypogammaglobulinemia, Fingolimod

## Abstract

Some people with multiple sclerosis (PwMS) exhibit reduced serum immunoglobulin (Ig) levels, potentially due to disease-modifying therapies (DMTs), which raises concerns about initiating anti-CD20 therapies. We assessed the frequency of hypogammaglobulinemia in PwMS who previously received non-anti-CD20 DMTs and evaluated short-term Ig level changes after switching to rituximab (RTX) or ocrelizumab (OCR). This retrospective study included PwMS starting RTX or OCR, with or without prior DMT exposure. Patients were grouped as treatment-naïve or receiving fingolimod (FING), natalizumab (NTZ), or moderate-efficacy DMTs (interferons, glatiramer acetate, dimethyl fumarate, or teriflunomide) before the switch. Among 417 included patients, 89 were treatment-naïve, 207 had received FING, 70 NTZ, and 51 moderate-efficacy DMTs. Before switching, hypogammaglobulinemia (IgG level <7 ​g/L) was rare in treatment-naïve and moderate-efficacy DMT groups (2 ​%) but more frequent after FING (29 ​%) and NTZ (14 ​%) treatment. One year after initiating RTX/OCR, IgG level slightly decreased in treatment-naïve patients (p ​< ​0.05), remained stable in NTZ and moderate-efficacy DMT groups, and increased significantly in FING-treated patients (8.0–8.6 ​g/L, p ​< ​0.0001), with a decline in hypogammaglobulinemia prevalence (29 ​%–21.5 ​%). FING exposure was associated with frequent IgG hypogammaglobulinemia, but switching to RTX/OCR was not linked to a short-term decrease in IgG level; instead, it led to a significant increase in level. These findings support that hypogammaglobulinemia should not be an absolute contraindication to switching to RTX/OCR after FING discontinuation given their efficacy in preventing MS reactivation. A secondary de-escalation strategy may be considered based on individual risk profiles and IgG level trajectories.

## Introduction

Hypogammaglobulinemia is a well-known adverse effect of anti-CD20 therapies in patients with multiple sclerosis (PwMS); it tends to worsen with prolonged anti-CD20 exposure and is associated with an increased risk of infection [[1], [2], [3], [4]]. Previous studies have suggested that other disease-modifying therapies (DMTs), particularly fingolimod (FING), may also be associated with risk of hypogammaglobulinemia [[5], [6], [7], [8], [9]].

Because of their high efficacy, anti-CD20 therapies are commonly used in PwMS with persistent disease activity despite prior DMTs or to prevent rebound disease activity in those discontinuing agents that limit lymphocyte trafficking [10,11]. Consequently, some patients with already reduced serum immunoglobulin (Ig) levels, potentially due to prior DMT exposure, may require initiation of anti-CD20 therapies for disease control. However, low baseline Ig levels is increasingly considered in treatment decision-making and may discourage the use of anti-CD20 therapies due to the risk of worsening immunosuppression.

The aim of the present study was to assess the frequency of hypogammaglobulinemia in patients receiving DMTs other than anti-CD20 agents as well as the short-term impact of initiating anti-CD20 therapy on Ig levels. We specifically sought to determine whether the introduction of anti-CD20 therapies could further lower pre-existing reduced Ig levels.

To address this question, we conducted a bicentric, retrospective study including all PwMS who initiated ocrelizumab (OCR) or rituximab (RTX), with or without prior exposure to another DMT, and for whom serum Ig levels were available immediately before the first OCR or RTX infusion.

## Methods

### Protocol and participants

This was a retrospective analysis of two structured data collections including PwMS who initiated RTX or OCR in two French expert MS centers after January 2015 until December 2023. Inclusion criteria were age >18 years; DMT-naïve before initiating RTX/OCR or received a DMT for at least 6 months, discontinued previous DMT no more than 3 months before the first infusion of RTX/OCR; and documented serum IgG and IgM levels measured within 3 months before the first infusion of RTX/OCR. Normal IgG and IgM levels were defined as ≥ 7 ​g/L and >0.4 ​g/L, respectively.

### Ethical approval

The authors obtained ethical approval from their institutional review boards (approval no.: PADS-21-60 for Marseille and DEC21-347 for Lille).

### Statistical analysis

Clinical characteristics and Ig levels were compared with Fisher's exact test for categorical variables and the Kruskal–Wallis test for continuous variables. When the Kruskal–Wallis test was significant, Dunn's post-hoc test was applied for pairwise group comparisons. The Wilcoxon signed-rank test was used to compare Ig levels measured within 3 months before RTX/OCR initiation and those measured from 6 months to 1 year after the switch, corresponding to the 6-month period following the second cycle. To assess risk factors for hypogammaglobulinemia, multivariate linear regression analyses were performed. The first model evaluated the odds of IgG level <7 ​g/L before RTX/OCR initiation and included type of prior DMT, age, sex, number of previous immunosuppressive DMTs (defined as all DMTs except glatiramer acetate [GA] and interferon beta-1a [IFNβ1a]), and Expanded Disability Status Scale (EDSS) score as covariates. The second model assessed the odds from 6 to 12 months after RTX/OCR initiation and included the same covariates, with the addition of anti-CD20 subtype (RTX vs. OCR). Odds ratios (ORs) and 95 ​% confidence intervals (CIs) were estimated. P ​< ​0.05 was considered statistically significant.

### Data availability

All data analyzed during this study will be shared anonymized by reasonable request of a qualified investigator to the corresponding author.

## Results

### Study population

A total of 560 patients initiated RTX/OCR treatment after January 2015: we excluded 52 because of a last treatment duration of <6 months and 22 because the duration of the last treatment was unknown. Among the remaining patients, 47 were excluded because the interval between the end of the last treatment and RTX/OCR initiation was >3 months, and 1 was excluded because of an unknown switch interval. We excluded an additional 21 patients because of missing serum Ig level data within 90 days before RTX/OCR initiation. Ultimately, 417 patients were included in the final analysis.

The demographic and clinical characteristics of the patients are in Table 1. At RTX/OCR initiation, the median (range) age was 41 (18–74) years, median disease duration 10 (0.06–37) years, and median EDSS score 3.0 (range: 0–8.5). A total of 89 patients were treatment-naïve before initiating RTX/OCR; 207 patients had previously received FING, 70 natalizumab (NTZ), and 51 moderate-efficacy DMTs such as GA, IFNβ1a, teriflunomide, or dimethyl fumarate. The median treatment duration was 5 (0.6–18), 2 (0.5–15), and 3 (0.5–16) years in the FING, NTZ, and moderate-efficacy DMT groups, respectively.

**Table 1:** Characteristics of patients.

Characteristic	Total (n ​= ​417)	Treatment-naïve (n ​= ​89)	Moderate-efficacy DMTs (n ​= ​51)	Natalizumab (n ​= ​70)	Fingolimod (n ​= ​207)
Sex, n (%)					
Female	284 (68)	59 (66)	36 (71)	45 (64)	144 (70)
Male	133 (32)	30 (34)	15 (29)	25 (36)	63 (30)
Age at RTX/OCR initiation, years, median (range)	41 (18–74)	39 (18–68)	40 (18–70)	43 (22–74)	42 (18–69)
Disease duration at RTX/OCR initiation, years, median (range)	10 (0.06–37)	2 (0.06–35) ∗∗∗	9 (0.7–32)	9 (0.8–32)	13 (0.8–37) ∗
Prior treatment duration, years, median (range)	NA	NA	3 (0.5–16)	2 (0.5–15)	5 (0.6–18) ∗∗∗
EDSS score at RTX/OCR initiation, median (range)	3 (0–8.5)	2 (0–7)	2 (0–7)	4 (0–7.5) ∗∗∗	3 (0–8.5) ∗∗

Statistical comparisons were performed with the moderate-efficacy DMT group as the reference, ∗P ​< ​0.05, ∗∗P ​< ​0.01, ∗∗∗P ​< ​0.001.

EDSS: Expanded Disability Status Scale; DMT: disease-modifying therapy; RTX: rituximab; OCR: ocrelizumab.

### Serum IgG/IgM levels before RTX/OCR

**Fig. 1:**
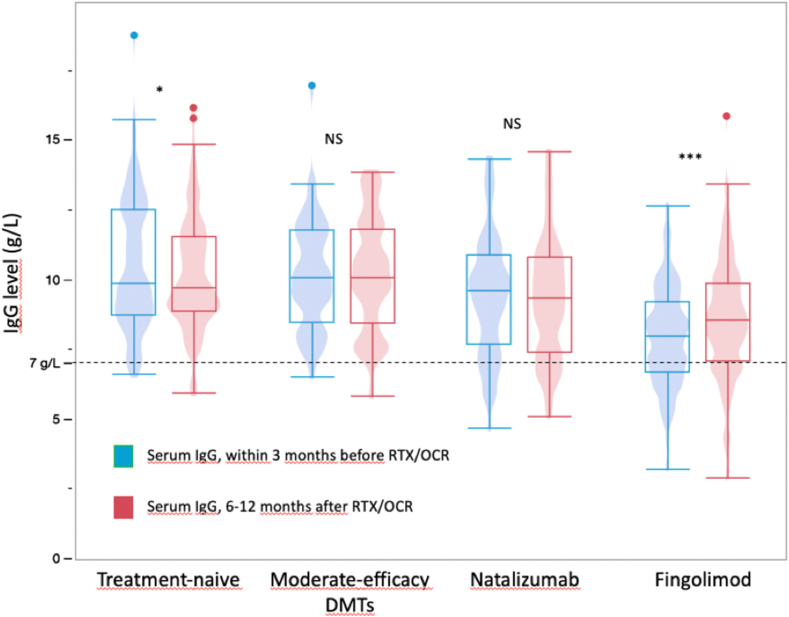
Evolution of serum IgG level before and after switching to RTX/OCR in the patient groups. IgG: gammaglobulin; RTX: rituximab; OCR: ocrelizumab.

Serum IgG/IgM levels within the 3 months before RTX/OCR initiation by patient group are in Table 2 and Fig. 1. Mean (SD) serum IgG level did not differ between patients who previously received moderate-efficacy DMTs and treatment-naïve patients (10.2 [2.0] vs 10.6 [2.4] g/L, *p* ​= ​1) and did not differ between patients who previously received NTZ and treatment-naïve patients (9.5 [2.2] vs 10.6 [2.4] g/L, *p* ​= ​0.08). However, mean serum IgG level was lower for patients who previously received FING than treatment-naïve patients (8.0 [1.8] vs 10.6 [2.4] g/L, *p* ​< ​0.001). Additionally, the proportion of patients with serum IgG level <7 ​g/L was higher among those who previously received FING and NTZ than treatment-naïve patients (29 ​% and 14 ​% vs 2 ​%, *p* ​< ​0.0001 and *p* ​< ​0.01, respectively).

**Table 2:** Proportion of patients with hypo-IgG before and after RTX/OCR initiation by patient group.

	Treatment-naïve (n ​= ​89)	Moderate-efficacy DMTs (n ​= ​51)	Natalizumab (n ​= ​70)	Fingolimod (n ​= ​207)
**Within 3 months before RTX/OCR**				
% Of patients with IgG level				
< ​7 ​g/L	2	2	14∗∗	29∗∗∗
< ​6 ​g/L	0	0	7∗	11∗∗∗
< ​4 ​g/L	0	0	0	1
**6**–**12 months after RTX/OCR**				
% Of patients with IgG level				
< ​7 ​g/L	3.5	2	17∗∗	21.5∗∗∗
< ​6 ​g/L	1	2	4.5	7∗
< ​4 ​g/L	0	0	0	0.5

Statistical comparisons were performed with the treatment-naïve group as the reference, ∗P ​< ​0.05, ∗∗P ​< ​0.01, ∗∗∗P ​< ​0.001.

IgG: gammaglobulin; RTX: rituximab; OCR: ocrelizumab.

On multivariate logistic regression analysis including type of prior DMT, age, sex, number of previous DMTs with immunosuppressive action and EDSS score, serum IgG level <7 ​g/L was associated with previous treatment with FING and NTZ versus treatment-naïve patients (OR 12.37, 95 ​% CI 2.74–55.68, *p* ​< ​0.005, and 6.19, 1.26–30.26, *p* ​< ​0.05) (Table 3). For patients who received FING, on multivariate logistic regression including age, sex, number of previous DMTs with immunosuppressive action, EDSS score and therapy duration, only treatment duration was associated with serum IgG level <7 ​g/L (OR ​= ​1.01, 95 ​% CI 1.003–1.017, *p* ​< ​0.005). For patients who received NTZ, multivariate logistic regression including the same variables did not identify any factor associated with IgG level <7 ​g/L.

**Table 3:** Factors associated with IgG level <7 ​g/L within 3 months before RTX/OCR initiation.

	IgG level <7 ​g/L within 3 months before RTX/OCR initiation	
	OR (95 ​% CI)	*p* Value
**Age (per year)**	1.01 (0.98–1.05)	0.31
**Sex (male vs female [ref.])**	1.47 (0.71–3.08)	0.30
**Number of previous immunosuppressive therapies**	1.16 (0.66–1.98)	0.58
**EDSS score**	1.03 (0.85–1.24)	0.73
**Last DMT before RTX/OCR initiation**		
Treatment naive [ref.]		
Moderate efficacy therapy	1.13 (0.09–12.97)	0.92
Natalizumab	6.19 (1.26–30.26)	0.02
Fingolimod	12.37 (2.74–55.68)	<0.005

OR: odds ratio; 95 ​% CI: 95 ​% confidence interval; IgG: gammaglobulin; EDSS: Expanded Disability Status Scale; DMT: disease-modifying therapy; RTX: rituximab; OCR: ocrelizumab.

Mean serum IgM level did not differ between patients who previously received moderate-efficacy DMTs and treatment-naïve patients (1.3 [0.6] vs 1.3 [0.6] g/L, *p* ​= ​0.67). The proportion of patients with serum IgM level <0.4 ​g/L did not differ between patients who previously received moderate-efficacy DMTs and treatment-naïve patients (0 ​% vs 0 ​%). Mean serum IgM level was lower in patients who previously received FING and NTZ than treatment-naïve patients (0.9 [0.50] and 0.77 [0.5] vs 1.3 [0.6] g/L, *p* ​< ​0.0001 for both comparisons). The proportion of patients with serum IgM level <0.4 ​g/L was higher for those who previously received FING and NTZ than treatment-naïve patients (8 ​% and 21.5 ​% vs 0 ​%, p ​< ​0.005 and p ​< ​0.0001, respectively).

The absence of events in the treatment-naïve group prevented the calculation of ORs for IgM level <0.4 ​g/L across treatment groups. For patients who received FING, on multivariate logistic regression including age, sex, number of previous DMTs with immunosuppressive action, EDSS score and therapy duration, no factors were associated with IgM level <0.4 ​g/L. For patients who received NTZ, on multivariate logistic regression including the same variables, only male sex was associated with IgM level <0.4 ​g/L (OR ​= ​5.8, 95 ​% CI 1.60–24.24, *p* ​< ​0.01).

### Serum IgG/IgM levels in the 6- to 12-month period after RTX/OCR initiation

Serum IgG and IgM levels in the 6- to 12-month period after RTX/OCR initiation by patient group are in Table 2 and Fig. 1. In the treatment-naïve group, mean IgG level significantly decreased between the 3 months before RTX/OCR initiation and the 6–12 months after initiation (10.6 [2.4] and 10.2 [2.0] g/L, respectively; *p* ​< ​0.05). The proportion of patients with IgG level <7 ​g/L was 2.0 ​% and 3.5 ​%, respectively. For patients who previously received moderate-efficacy DMTs, mean IgG level remained stable (10.2 [2.0] and 10.3 [2.0] g/L, respectively; *p* ​= ​0.79). The proportion of patients with IgG level <7 ​g/L was 2.0 ​% at both time points. Patients who previously received NTZ exhibited no significant change in mean IgG level between the two periods (9.5 [2.2] and 9.4 [2.2] g/L, respectively; *p* ​= ​0.34). The proportion of patients with IgG level <7 ​g/L was 14 ​% and 17 ​%, respectively. For patients who previously received FING, mean IgG level significantly increased between the two periods (8.0 [1.8] and 8.6 [2.0] g/L, respectively; *p* ​< ​0.0001). The proportion of patients with IgG level <7 ​g/L was 29.0 ​% and 21.5 ​%, respectively.

On multivariate logistic regression analysis including type of prior DMT, age, sex, number of previous DMTs with immunosuppressive action, EDSS score and type of anti-CD20 (RTX vs OCR), only previous treatment with FING was associated with serum IgG level <7 ​g/L (OR 6.67, 95 ​% CI 1.82–24.41, p ​< ​0.01) (Table 4).

**Table 4:** Factors associated with IgG level <7 ​g/L at 6–12 months after RTX/OCR initiation.

	IgG level <7 ​g/L at 6–12 months after RTX/OCR initiation	
	OR (95 ​% CI)	*p* Value
**Age (per year)**	1.01 (0.97–1.05)	0.59
**Sex (male vs female [ref.])**	1.53 (0.73–3.22)	0.25
**Number of previous immunosuppressive therapies**	1.32 (0.76–2.26)	0.31
**EDSS score**	1.14 (0.92–1.41)	0.21
**Type of anti-CD20 (RTX vs OCR [ref.])**	2.21 (0.95–5.13)	0.065
**Last DMT before RTX/OCR initiation**		
Treatment naive [ref.]		
Moderate efficacy therapy	0.86 (0.08–8.89)	0.91
Natalizumab	3.67 (0.92–14.52)	0.02
Fingolimod	6.67 (1.82–24.41)	<0.01

OR: odds ratio; 95 ​% CI: 95 ​% confidence interval; IgG: gammaglobulin; EDSS: Expanded Disability Status Scale; DMT: disease-modifying therapy; RTX: rituximab; OCR: ocrelizumab.

In the treatment-naïve group, mean IgM level significantly decreased between the 3 months preceding RTX/OCR initiation and the 6–12 months after initiation (1.3 [0.6] and 0.9 [0.5] g/L, respectively; *p* ​< ​0.0001). The proportion of patients with IgM <0.4 ​g/L was 0 ​% and 14.5 ​%, respectively. For patients who previously received moderate-efficacy DMTs, mean IgM level significantly decreased (1.3 [0.6] and 1.0 [0.5] g/L, respectively; *p* ​< ​0.0001). The proportion of patients with IgM level <0.4 ​g/L was 0 ​% and 6 ​%, respectively. For patients who previously received NTZ, mean IgM level significantly decreased (0.7 [0.5] and 0.6 [0.4] g/L, respectively; *p* ​< ​0.005). The proportion of patients with IgM <0.4 ​g/L was 21.5 ​% and 38.5 ​%, respectively. For patients who previously received FING, mean IgM level significantly decreased between the two periods (0.9 [0.5] and 0.8 [0.5] g/L, respectively; p ​< ​0.0001). The proportion of patients with IgM level <0.4 ​g/L was 8 ​% and 17 ​%, respectively.

On multivariate logistic regression analysis including type of prior DMT, age, sex, number of previous DMTs with immunosuppressive action, EDSS score and type of anti-CD20 (RTX vs OCR) as independent variables, previous treatment with NTZ and RTX was associated with serum IgM level <0.4 ​g/L (OR 2.99, 95 ​% CI 1.30–6.84, p ​< ​0.01 and 2.15, 1.11–4.16, p ​< ​0.05).

## Discussion

This study provides evidence of the frequency of hypogammaglobulinemia in patients who received DMTs other than anti-CD20 as well as the short-term impact of switching to anti-CD20 therapies on Ig levels. The risk of hypogammaglobulinemia was associated with treatment with FING and NTZ in PwMS. FING treatment was primarily linked to a risk of reduced IgG level, whereas NTZ treatment was more strongly associated with a risk of reduced IgM level. In contrast, moderate-efficacy DMTs, including GA, IFNβ1a, dimethyl fumarate, and teriflunomide, were not associated with a risk of hypogammaglobulinemia as compared with DMT-naïve patients. Shortly after switching to anti-CD20 therapy, mean IgG level significantly increased in patients who previously received FING, with no significant change in those who previously received NTZ. By contrast, mean IgM level decreased regardless of prior therapy.

Previous studies have reported similar findings in PwMS who received FING, NTZ, or moderate-efficacy DMTs [5,7,8]. In our study, although both FING and NTZ were associated with a risk of reduced Ig levels, FING was primarily associated with reduced IgG level, whereas NTZ was more strongly associated with reduced IgM level. In contrast, patients who received moderate-efficacy DMTs showed a lower extent of Ig level changes. In the present study, approximately 30 ​% of patients who received FING for a mean of 6 years had IgG level <7 ​g/L. Of note, the duration of therapy was associated with a risk of reduced IgG level. FING, which inhibits the egress of lymphocytes from secondary lymphoid tissues, induces a chronic reduction in B-cell levels that may lead to decreased Ig levels [[12], [13], [14], [15], [16]]. The mechanisms underlying reduced Ig levels associated with NTZ remain unclear. NTZ blockade of CD49d/VLA-4 is associated with increased release and reduced homing of B cells in bone marrow and spleen, which may explain the increased frequency of B cells in the blood. The frequency of blood plasmablasts, B cells particularly involved in Ig synthesis, is decreased in patients who receive NTZ [17,18]. Nevertheless, the reason for the greater impact of NTZ on IgM rather than IgG level remains unknown.

The present study was primarily designed to assess the outcomes of serum Ig levels in patients with prior DMT-associated hypogammaglobulinemia after initiating RTX/OCR, therapies known to particularly reduce Ig levels. This question is of major importance because previously RTX/OCR was found associated with the lowest risk of disease reactivation after switching from FING or NTZ. However, as discussed earlier, we have shown that both FING and NTZ are associated with significant odds of hypogammaglobulinemia. Regarding the evolution of IgG level after the switch from FING, the findings of the present study are partly reassuring. In patients switching from FING, mean serum IgG level increased in the years after RTX/OCR initiation, and the proportion of patients with reduced IgG level (<7 ​g/L) decreased from 30 ​% to approximately 20 ​%. The increase in IgG level could be related to enhanced plasma cell trafficking after discontinuation of FING, without a reduction in plasma cell numbers after RTX/OCR initiation, because these cells do not express CD20. We observed a significant decrease in the evolution of IgM level after the switch from FING. However, the proportion of patients with reduced IgM level (<0.4 ​g/L) in the year after RTX/OCR initiation was similar to that in the treatment-naïve group, affecting about 15 ​% of patients. This decrease in IgM level could be related to the rapid impact of RTX/OCR on number of plasmablasts, which express CD20 to a greater extent and are more involved in IgM synthesis.

These findings support that anti-CD20 therapies should remain a therapeutic option after FING discontinuation, even in patients with low IgG level, given their efficacy in preventing MS reactivation. However, a subsequent de-escalation strategy may be considered to mitigate a further decline in IgG associated with repeated anti-CD20 infusions.

For patients switching from NTZ, we found no significant change in IgG level within the first year after RTX/OCR initiation. The proportion of patients with reduced IgM level (<0.4 ​g/L) increased from 21 ​% to 38 ​% during the year after RTX/OCR initiation. As previously discussed, this decrease in IgM level after switching to anti-CD20 therapies could be related to the rapid impact of these treatments on plasmablasts.

Previous studies have shown that in patients receiving RTX/OCR, reduced IgG level rather than IgM level could more strongly be associated with risk of infection [[1], [2], [3]]. In this context, our findings are reassuring regarding the short-term disease evolution because they suggest that initiating RTX/OCR in individuals with reduced IgG level secondary to FING or NTZ treatment is not associated with any further immediate decline in IgG level. On the contrary, patients who previously received FING showed a significant increase in IgG level. Nevertheless, as demonstrated in numerous studies, a progressive decrease in IgG level may still be expected with continued RTX/OCR therapy [1,2,9].

The present study suggests that in PwMS requiring discontinuation of FING or NTZ, the presence of hypogammaglobulinemia should not be considered an absolute contraindication to switching to RTX/OCR. This is particularly important given that anti-CD20 therapies have demonstrated the highest efficacy in preventing disease reactivation in this high-risk switching context. A secondary de-escalation strategy may be considered, depending on individual patient risk and Ig level trajectory [19]. Further studies with larger samples are required to confirm these findings.

## Contributions for the study

Marine Perriguey played a major role in the acquisition of the data, gathering of data and writing the manuscript.

Camille Rigollet played a major role in the acquisition of the data, gathering of data and writing the manuscript.

Sean A. Freeman played a major role in the acquisition of the data, gathering of data and writing the manuscript.

Lisa Graille-Avy played a major role in the acquisition of the data and the gathering of data.

Jean-Christophe Lafontaine played a major role in the acquisition of the data.

Bruno Lemarchant played a major role in the acquisition of the data.

Tifanie Alberto played a major role in the acquisition of the data.

Sarah Demortière played a major role in the acquisition of the data.

Clémence Boutiere played a major role in the acquisition of the data.

Audrey Rico played a major role in the acquisition of the data.

Frédéric Hilézian played a major role in the acquisition of the data.

Pierre Durozard played a major role in the acquisition of the data.

Jean Pelletier played a major role in the acquisition of the data.

Adil Maarouf played a major role in the acquisition of the data.

Hélène Zéphir planned and conducted the study, and wrote the manuscript.

Bertrand Audoin planned and conducted the study, and wrote the manuscript.

## Funding/Support

This observational study was not supported by any specific funding.

## Declaration of competing interest

The authors declare that they have no known competing financial interests or personal relationships that could have appeared to influence the work reported in this paper.
